# Shining a light on transition metal chalcogenides for sustainable photovoltaics

**DOI:** 10.1039/c7sc00642j

**Published:** 2017-03-13

**Authors:** Peter D. Matthews, Paul D. McNaughter, David J. Lewis, Paul O'Brien

**Affiliations:** a School of Chemistry , University of Manchester , Oxford Road , Manchester , M13 9PL , UK . Email: paul.o'brien@manchester.ac.uk; b School of Materials , University of Manchester , Oxford Road , Manchester , M13 9PL , UK

## Abstract

Transition metal chalcogenides are an important family of materials that have received significant interest in recent years as they have the potential for diverse applications ranging from use in electronics to industrial lubricants. One of their most exciting properties is the ability to generate electricity from incident light. In this perspective we will summarise and highlight the key results and challenges in this area and explain how transition metal chalcogenides are a good choice for future sustainable photovoltaics.

## Abstract 2

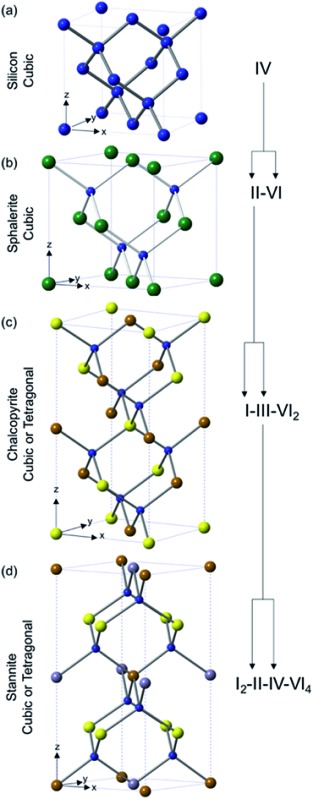
Transition metal chalcogenides are an important family of materials that have received significant interest in recent years as they have the potential for diverse applications ranging from use in electronics to industrial lubricants.

## Introduction

Transition metal chalcogenides (TMCs) are a class of materials that have seen a huge surge in interest in the past few years, with many researchers focusing on their exciting properties and extensive range of applications including solar cells, sensors, field effect transistors and water splitting photocatalysis.^1^ A number of transition metal chalcogenides adopt a layered structure that bestows chemical and electronic properties that differ to those of bulk semi-conductors.^1^ Part of the boom in interest in this area has been driven by the rise of two-dimensional materials, and modern synthetic methods that can be used to synthesise monolayers of these materials from a ‘bottom up’^2^ or ‘top down’^3^ approach. A number of other authors have offered comprehensive reviews on this area and we would like to direct the reader's attentions to these.^4–8^

The other reason for the rise in interest in TMCs, and the focus of this perspective, is the photovoltaic (PV) potential of this class of compounds, which in some cases are cheap, earth abundant and non-toxic and therefore offer singular opportunities for sustainable energy production. The main advantage that TMCs offer over other mainstream PV materials such as organic photovoltaics (OPVs) and lead perovskites is a greater stability. OPVs suffer from bleaching, which is where oxygen reacts with the organic molecules that form the photoabsorber and oxidises them.^9^ Lead perovskites have a similar stability issue, with the material being sensitive to oxygen and water.^10,11^

Classical TMC photovoltaics centred around the Cd(S,Se) family, whilst second generation materials feature indium, gallium or arsenic. The use of cadmium has been subject of strict international sanctions limiting its industrial applicability. Equally, concerns still abound about the worldwide supply and sustainable international availability of In, Ga and As^12^ has fuelled interest into other chalcogenide materials.

Photovoltaic devices are regularly cited as sources of ‘green energy’, but for them to be truly sustainable and economically viable the cost of the material must be low and the efficiency of the device high. In 2009 Wadia *et al.* modelled the annual potential energy production of a series of photovoltaic materials and plotted this against the material production costs.^13^ They found that materials such as FeS_2_, Cu_2_S and Cu_2_ZnSnS_4_ have the greatest energy production potential as a function of material cost, so the challenge at this present juncture is to realise the full potential of these materials. Note that the 2017 Materials Commodity Survey by the US Geological Survey^14^ indicates that the cost of materials hasn't substantially changed since 2009 and so the results of the Wadia report are still relevant.

**Fig. 1:**
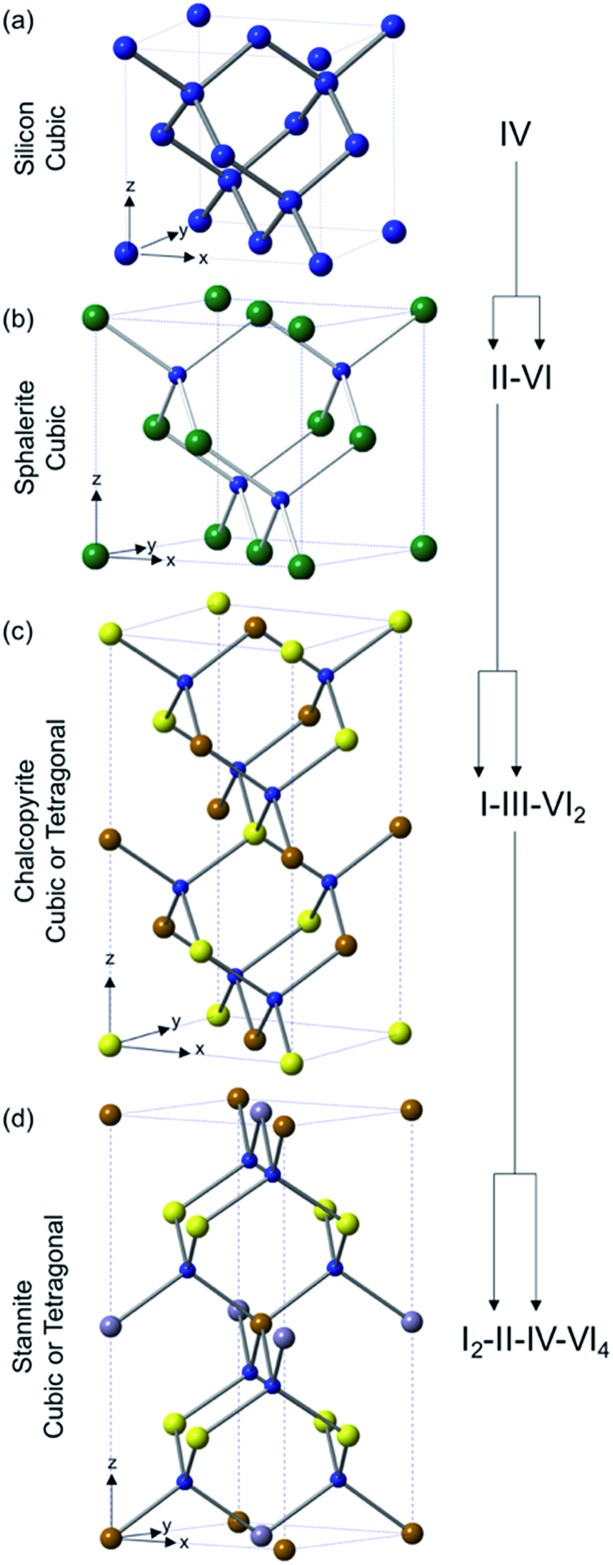
An illustration of the evolving structural relationship between (a) IV (diamond structure, cubic *Fd*3*m*), (b) binary, M_*x*_E_*n*_, II–VI (zinc blende structure, cubic *F*43*m*), (c) ternary, M_*x*_M′_*y*_E_*n*_, I–III–VI_2_ (chalcopyrite structure, tetragonal *I*42*d*, though can also be cubic) and (d) quarternary, M_*x*_M′_*y*_M′′_*z*_E_*n*_, I_2_–II–IV–VI_4_ (stannite structure, tetragonal *I*42*m*, though can also be cubic) semiconductor materials. Blue = S, green = Zn, yellow = Cu, brown = Fe, grey = Sn.

TMC semi-conductors that are suitable for photovoltaic devices cover a staggeringly large range of materials (at least 15 000 different compounds according to a search of the ICSD). These classes can be broken down into three main categories: binary (M_*x*_E_*n*_), ternary (M_*x*_M′_*y*_E_*n*_) and quaternary (M_*x*_M′_*y*_M′′_*z*_E_*n*_) (where M = transition metal, M′/M′′ = transition or other metal and E = S, Se or Te) systems (Fig. 1). The classes are often described in Roman numerals, with the numeral referring to the oxidation state of the metal and the group of the chalcogen or pnictogen, *e.g.* II–VI (CdS), I–III–VI_2_ (CuInS_2_) or III–V (InP).

**Fig. 2:**
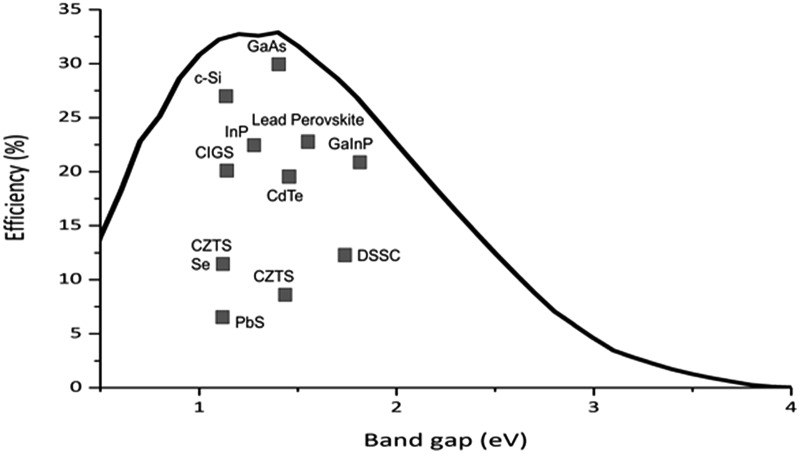
An illustration of the Shockley–Quessier limit (maximum theoretical efficiency) for solar cells under AM 1.5 illumination. The band gaps of a selection of photovoltaic materials is shown for comparison.

One requirement for a good photoactive semiconductor is that it must have a band gap between 1.0–1.5 eV between the lower lying valence band and the higher energy conduction band. This is a consequence of the Shockley–Quessier (SQ) limit, which is the theoretical maximum efficiency for a single pn-junction solar cell. Fig. 2 shows the SQ limit for semiconductors under the standard atmospheric solar emission spectrum (with an air mass of coefficient of 1.5 and a solar zenith angle of 48.19°, *i.e.* AM 1.5), demonstrating that the maximum photoconversion efficiency lies in the 1.0–1.5 eV region.^15^

The principles behind the photoactivity of TMC semiconductors can be understood by considering layered binary systems. Group IV dichalcogenides, such as ZrS_2_ and HfS_2_, have an analogous electronic structure to TiO_2_, *i.e.* a valence band derived from S-p orbitals and a conduction band of Zr/Hf-d orbitals. For the other TMCs the metal has electrons in the d-orbitals: these occupy states that lie between the E-p orbital and the empty M-d orbitals. For the ternary and quaternary systems that do not adopt a layered structure, an appropriate example is CZTS (Cu_2_ZnSnS_4_). In this case the upper valence band consists of hybridised S-p and Cu-d orbitals, while the conduction band is derived from the hybridisation of S-s/p and Sn-s orbitals.^16^

TMC semiconductors are beginning to realise their potential as building blocks in photovoltaic devices based on thin films,^17^ quantum dots (QD),^18^ and dye-sensitized solar cells (DSSC).^19^ One of the major challenges that face researchers is to find a semiconductor material with a band gap in the range 1.0–1.5 eV and with a high absorption coefficient that can be made cheaply from elements that are plentiful. Fortunately there are many choices of TMC that fit these criteria, though some have shortcomings that will be discussed below. This perspective will seek to highlight the key TMCs that offer the greatest potential for commercialisation and will discuss the properties and synthetic routes toward these materials.

## Synthetic routes to devices

Transition metal chalcogenides have found a number of uses in PV devices, ranging from photoabsorber layers to buffer layers and anodes in DSSCs. The form they take in these devices is generally either as a nanostructured thin film or as quantum dots.

The manufacture of thin films can be split into three rather broad sections: chemical vapour deposition, atomic deposition or solution processing. These broad sections encompass a wide variety of techniques that have each earned an acronym in their own right, but can be collated based on a basic similar principle.

Chemical vapour deposition (CVD), in all its major forms including metal–organic CVD (MO-CVD), and low pressure CVD, (LP-CVD) involves the decomposition of a precursor in the gas phase leading to the growth of a substrate on a target. The CVD process has been used extensively by researchers to generate high quality thin films.^20–24^ The range of suitable precursors is large, with many options available as to whether individual components or single source precursors are used. Techniques such as aerosol assisted CVD (AA-CVD) have been developed to circumvent the need for volatile precursors and as such widens the scope of precursors available for materials fabrication.^25^

Atomic deposition encompasses a broad range of techniques, but the purpose of these in general is to deposit a layer of the individual components that are then later annealed in the presence of elemental chalcogen. The deposition techniques include atomic layer deposition (ALD),^26^ successive ionic layer adsorption and reaction (SILAR),^27^ sputtering^28^ and pulsed laser deposition.^29^ The advantages of processes such as ALD are precise thickness control at the monolayer level, which can be important for optimising device performance. The disadvantages of these processes are that they are not suitable for manufacturing large scale devices, often require ultra-high vacuum with the associated complications, and at the laboratory level are often custom builds, leading to difficulties in reproducing results from one instrument to another.

Solution processing of TMCs can include the synthesis of nanocrystalline material, which may be treated as an ‘ink’ and processed into a film,^30,31^ or processes such as chemical bath deposition (CBD).^32,33^ Nanocrystalline TMCs have been synthesised by the ubiquitous hot-injection route, which has proven to be applicable to binary, ternary and quaternary systems, as well as others such as solvothermal syntheses.

## Binary systems

Binary transition metal chalcogenides have the form M_*x*_E_*n*_ (M transition metal, E = S, Se, Te). There are a vast range of binary TMC systems that have demonstrate suitable properties for photovoltaic systems, including: FeS_2_,^34,35^ CdS,^36^ Cu_*x*_S,^37,38^ CuSe,^39^ MoS_2_,^40,41^ RuS_2_,^42–45^ ZrS_2_/Se_2_,^46^ TaS_2_ (ref. 47) and AgS.^48^

Amongst these systems Cd(S,Se), FeS_2_ and the various copper sulfides demonstrate the most exciting properties and are perhaps the most well-known.

Cadmium sulfide/selenide quantum dots were almost ubiquitous in the early 2000s, as facile routes has been developed for their synthesis in the preceding decade^49–51^ and had ideal photoelectric properties. The electronic properties of the Cd(S,Se) quantum dots, notably the band gap, can be easily tuned by controlling the proportion of sulfur/selenium.^52^ However, cadmium has a well-documented high toxicity,^53,54^ which has led to strict EU regulation. This in theory limits the suitability of cadmium chalcogenides as a photovoltaic for anything other than a laboratory scale test. Cd solar cells are however still being developed and in 2016 First Solar set a new record efficiency of 22.1% for CdTe with a thin film device.^55^

**Fig. 3:**
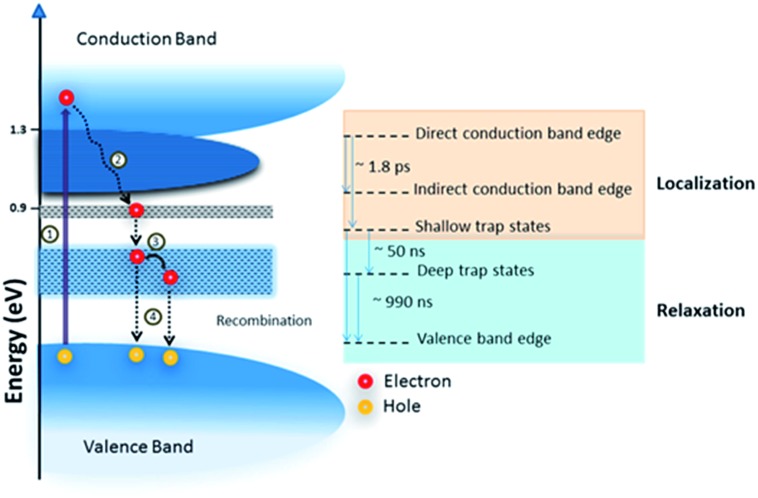
Origin of the loss of charge carriers in FeS_2_. (1) An electron is optically excited from the valence to the conduction band, (2) the charge carrier is rapidly localized at the indirect band edge and low lying shallow defect states, (3) slower electron relaxation to long lived trap states, and (4) electron–hole recombination. Reprinted with permission from ref. 35, ©2016 American Chemical Society.

FeS_2_, pyrite (or Fool's Gold) has a band gap of 0.95 eV and an absorption coefficient of 10^5^ cm^–1^. Combining this with the extremely low raw material costs and simplicity of synthesis^56^ at face value pyrite should make an excellent candidate for PV devices. Indeed, nanostructured FeS_2_ has been used in DSSCs, as a photoconductor, in a p–i–n heterojunction and in bulk heterojunction inorganic–organic hybrid solar cells.^57–60^ However, it seems that surface defects brought upon by sulfur vacancies can severely affect the electronic properties. Steinhagen *et al.* have shown that nanocrystal devices are particularly prone to this owing to the high concentration of grain boundaries and presumably the high fraction of atoms that reside at the surface in nanoscale particles.^34^ Shukla *et al.* demonstrated that photovoltages can be obtained from pyrite nanocubes by sulfurizing a deposited colloidal ink. They conclude that surface defects are the major contribution to hole–electron recombination (Fig. 3) and increased efficiency may be obtained either by reducing the grain boundaries or reducing defects through an improved synthetic route.^35^

Ruthenium sulfide adopts the same pyrite structure as FeS_2_, and like iron sulfide, has an appropriate (though indirect) band gap of 1.3 eV. Single crystals of RuS_2_ have been shown to oxidise H_2_O upon illumination, but it is thought that RuO_2_ is probably responsible.^42^ The single crystals show a limited photocurrent, but deposited thin films do not, possibly due to a high electron/hole re-trapping and combination rate.^44,45^

Copper sulfide exists in a large number of phases related to the stoichiometry of Cu_*x*_S, which all have a band gap in the region of 1.2–2.0 eV.^37^ For *x* < 2, the band gap is closer to 2.0 eV and so these are of limited use in PV applications, however Cu_2_S is an indirect band gap semiconductor with a bulk band gap of 1.21 eV,^38^ its selenide analogue, Cu_2_Se, has an indirect band gap of 1.4 eV.^39^ Cu_2_S was widely used in combination with CdS in the 1960s–1980s^61^ but diffusion of Cu^+^ ions into the CdS layer degraded the PV cell over time. Wu *et al.* reported the synthesis of Cu_2_S nanocrystals from the reaction of copper(ii) acetylacetonate and ammonium diethyldithiocarbamate in a mixed solvent of dodecanethiol and oleic acid. They then spin coated these nanocrystals onto a layer of CdS nanorods to produce a PV device of with an efficiency of 1.6%.^38^ Mousavi-Kamazani have since deposited it on TiO_2_ as part of a DSSC to improve the efficiency to 8.3%.^62^

Also in group 11, both AgS and Ag_2_S have band gaps ∼1.1 eV,^48,63^ which should give them both appealing PV characteristics. However, few serious attempts have been made to optimise a silver sulfide based solar cell. Tubtimtae^63^ and Shen^64^ have both tested devices, with the former achieving an efficiency of 1.70%.

MoS_2_ is a layered TMC that has seen a substantial amount of recent research owing to its ability to exist in monolayer form.^1^ It has a direct band gap of 1.85 eV in its monolayer and an indirect band gap of 1.2 eV in the bulk. The band gap is related to the number of layers of MoS_2_ and so it is a strong candidate for PV applications, as the band gap can be tuned by controlling the thickness.^65^ Gourmelon *et al.* and Shanmugam *et al.* have both reported the use of MoS_2_ in solar cells to give an efficiency of 1.3%,^40,66^ whilst Gong *et al.* have shown that the band gap can be further tuned by the introduction of selenium.^41^ One potential problem for molybdenum sulfide in the thermodynamically stable 2H–MoS_2_ structure is that poor alignment of the layers can drastically reduce the photosensitivity. The Van der Waals planes between the monolayer sheets of 2H–MoS_2_ contain a high concentration of defects, which can trap charge carriers. If a high photoconversion efficiency is to be achieved then these must be reduced. Indeed, efforts have recently been directed to ameliorate these defects using the organic superacid bis(trifluoromethane)sulfonamide, which led to the almost complete suppression of non-radiative recombination and a photoluminescence quantum yield improvement from 0.6% to 95%, which paves the way to the use of 2D MoS_2_ in photovoltaic devices.^67^ This approach has been extended to WS_2_, though does not work for MoSe_2_ or WSe_2_.^68^

The ZrS_*x*_Se_2–*x*_ family has been grown by Moustafa *et al.*, and the band gap range found to be 1.18 eV (ZrSe_2_) to 1.7 eV (ZrS_2_).^46^ This suggests that zirconium selenide should be tested for PV characteristics, though to the best of our knowledge this has not been carried out.

Manganese(ii) sulfide has been used as a dopant in PbS, CdS, CdSe and ZnS quantum dots,^69^ with a PV conversion efficiency of 4.25% demonstrated in a PbS quantum dot DSSC by Punnoose *et al.* ^70^ It has not, however, been used as the photoabsorber by itself as it has a band gap of 3.1 eV.^71^

**Fig. 4:**
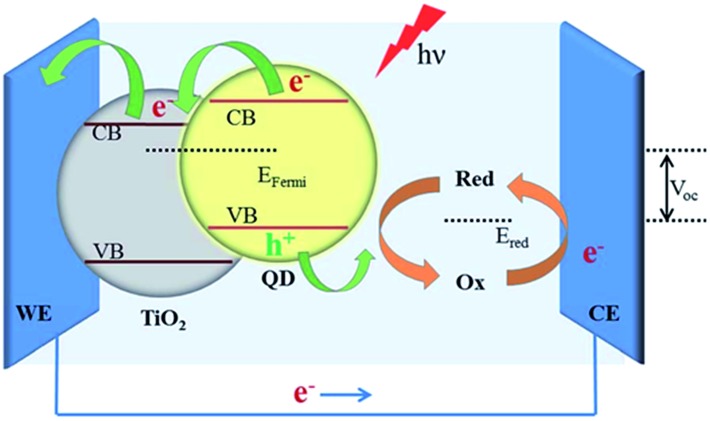
A schematic of a quantum dot (QD) sensitized solar cell (QDSSC). The QD is photoexcited and transfers an electron to TiO_2_ on an indium tin oxide (ITO) working electrode (WE). The electrolyte undergoes a redox cycle on the counter electrode (CE) for which metal chalcogenides have been proposed. Reprinted with permission from ref. 19, ©2015 Royal Society of Chemistry.

Typically a DSSC has a platinum counter electrode (Fig. 4), and a significant amount of research has been directed to reducing this reliance on noble metals. Naturally, some attention has been bestowed on carbon materials (graphene, nanotubes, carbon black),^72^ but molybdenum,^73^ nickel^74^ and cobalt sulfides^75^ have also been investigated. In a related manner, tantalum sulfide nanosheets with a band gap of 1.92 eV are promising candidates for electrodes in organic photovoltaic (OPV) devices.^47^

On a final note, a main group binary chalcogenide that has attracted interest for photovoltaics is tin monosulfide (SnS), due to its band gap commensurate with solar absorption (typically 1.1–1.4 eV),^76^ with a theoretical power conversion efficiency of up to 24%. Gordon and co-workers demonstrated a PV cell with record efficiency of 4.4%,^77^ and thus there is great room for improvement. Efforts in our group have focused on using AACVD to fabricate these semiconductors as thin films suitable for eventual use in PV device architectures.^78–80^ Interestingly, SnS is a Van der Waals layer structure, and we have shown that thinning these materials to the 2D limit can control the band gap energy in a predictable manner that is layer dependent in nature.^81^

## Ternary

**Fig. 5:**
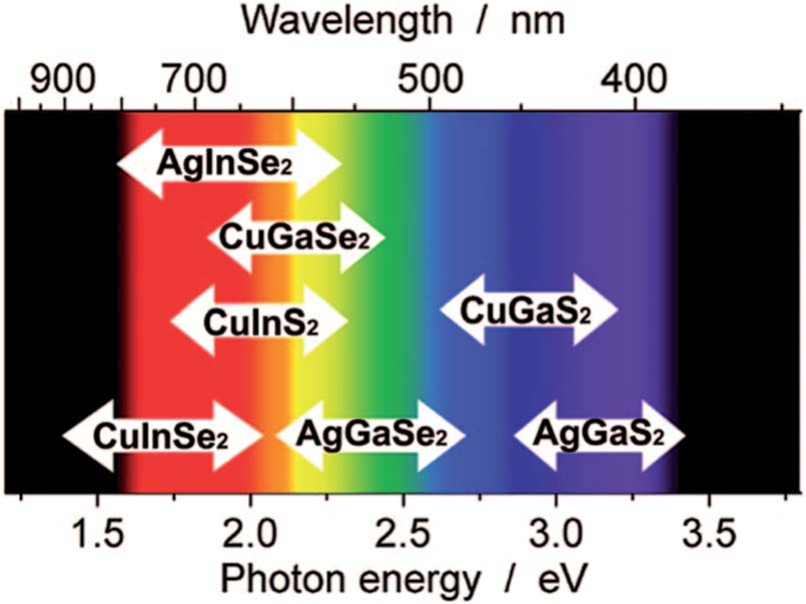
An illustration of the visible spectrum and where some typical chalcopyrite-type I–III–VI_2_ nanocrystals absorb when between 2 and 5 nm in size. Reprinted with permission from ref. 82, ©2009 American Institute of Physics.

When the metal of a binary metal chalcogenide system is substituted for two metals providing the same total charge and hence is isoelectric, *i.e.* moving from M_*x*_E_*n*_ (Fig. 1b) to M_*x*_M′_*y*_E_*n*_ (Fig. 1c), a new category of ternary metal chalcogenides is accessed. The use of two different metals allows access to the band gaps and not accessible to binary metal chalcogenides. A common ternary system possesses two metals each with oxidation states of +1 and +3 in combination with a pair of chalcogens each in oxidation state –2. This is described as a I–III–VI_2_ *e.g.* CuInS_2_. The parent binary system to I–III–VI_2_ is the II–VI system where CdSe is an example (Fig. 1). Systems where there are two different chalcogens, *i.e.* M_*x*_M′_*y*_E_*n*_E′_*m*_, also fall under the general I–III–VI_2_ system and are classified as ternary systems despite containing four separate elements. In an identical fashion to their parent binary systems, ternary systems also undergo quantum confinement and behave as quantum dots.^82^ Utilising the energy modulation effects by manifestation of quantum confinement in these materials allows ternary metal chalcogenides to access the entire solar spectrum which is highly beneficial for light harvesting and makes them an attractive alternative to toxic binary metal chalcogenides like cadmium chalcogenides (Fig. 5).

**Fig. 6:**
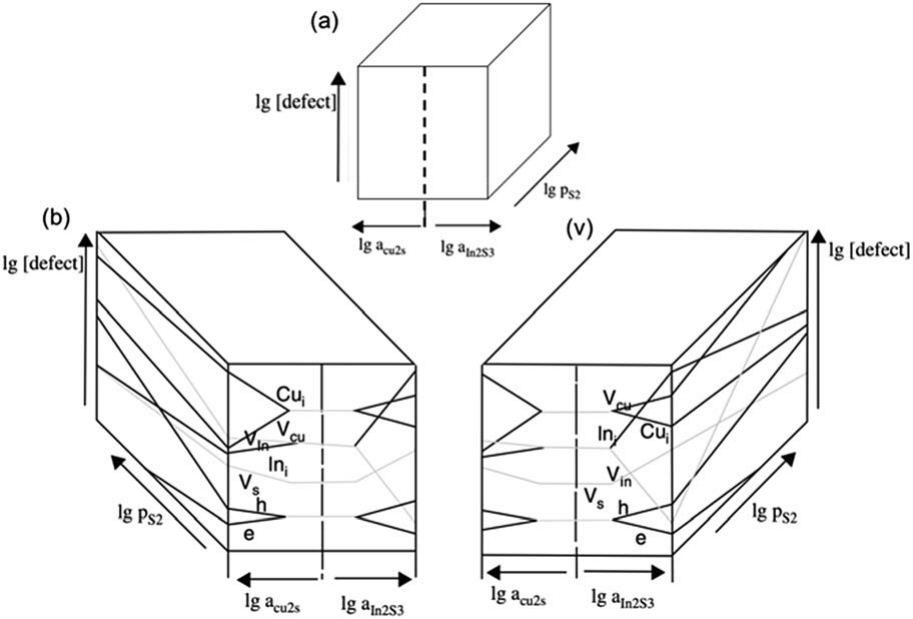
(a) The 3D concept of a Brouwer diagram for CIS, *i.e.* how a change in stoichiometry (in Cu, In or S) results in defects in (b) a Cu-rich regime and (c) an In-rich regime. Adapted with permission from ref. 88, ©2008 Springer.

The chalcopyrite phase of copper indium sulfide, CuInS_2_ (often abbreviated to CIS) is a ternary metal chalcogenide that has been explored as a component of heterojunction PV devices. CuInS_2_ is a useful material for use in photovoltaic devices due to a direct band gap of 1.5 eV, an absorption coefficient >10^5^ cm^–1^, tolerance to defects and high radiation hardness.^83^ Early devices used CIS in combination with CdS or InP and homojunction devices in the 1970's.^84–86^ The growth conditions of the CuInS_2_ and resulting defects also govern if it is an n- or p-type semiconductor, depending on if it is formed in an indium or sulfur rich environment respectively (Fig. 6).^87,88^ The high proportion of defects also leads to advantageous properties such as the ability to take a high loading of dopants and band gap tuning through the number of defect sites.^89,90^ Although useful, these properties can also lead to compositional differences between nanocrystals of identical size within a batch leading to broadening on the ensemble properties such as the luminescence peak of colloidal nanocrystals.^91^

**Fig. 7:**
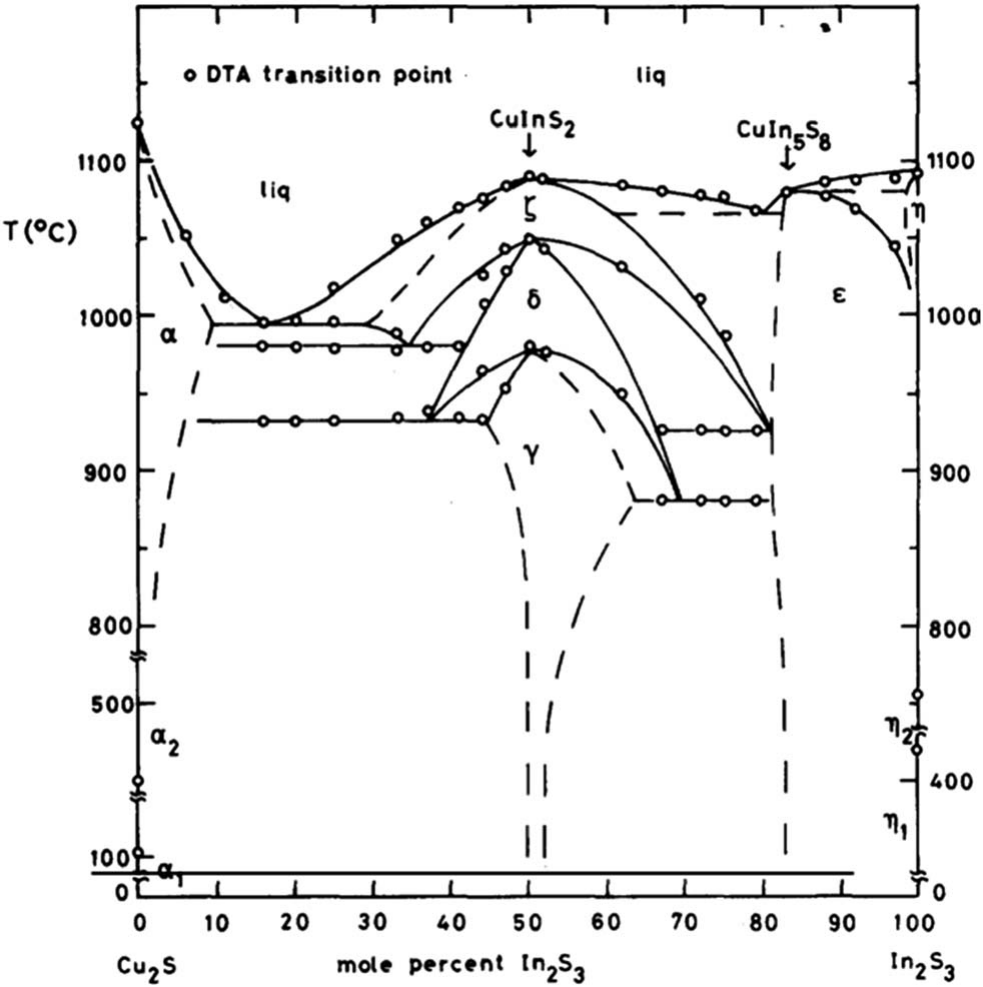
Tentative diagram of the *T*–*x* relations along the join Cu_2_S–In_2_S_3_ at moderate pressure(s). The single phase regions are indicated by their respective symbol. The two phase regions, which lie in between the single phase regions are not indicated. The gamma phase is the target CuInS_2_ chalcopyrite phase. Reprinted with permission from ref. 88, ©1980 Elsevier.

The difficulty in forming phase pure CuInE_2_ lies in that Cu(i) is a soft Lewis acid whereas In(iii) is a hard Lewis acid. As a consequence their reactivity towards the sulfur precursor, often a Lewis base, is different. Accordingly the formation of Cu_*x*_S_*y*_ phases is a common observation whilst optimising synthetic routes.^92^ Thus balancing the reactivity of the precursors used at the same time complicates the optimisation of the synthetic strategies towards CuInS_2_. The resulting nanocrystals often differ from the ideal C : I : S elemental ratio of 1 : 1 : 2 thus allowing another degree of control over the properties of the nanocrystals produced. The phase diagram of CuInS_2_ is complex and at below 800 °C the window to form CuInS_2_ is narrow (Fig. 7).^93^

**Fig. 8:**
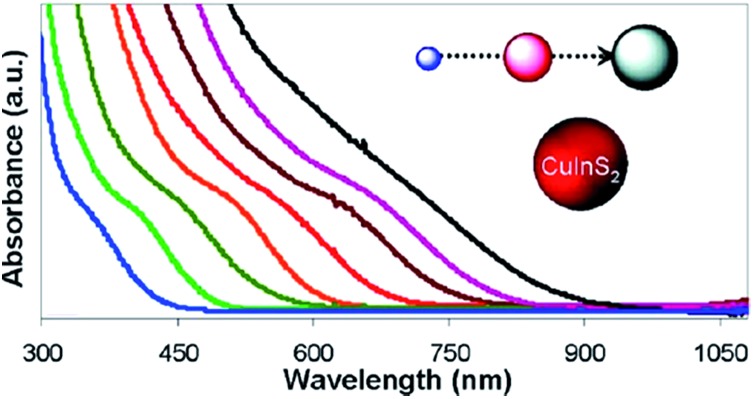
Absorption spectra of CIS nanocrystals of different sizes between 2 and 16 nm reprinted with permission from ref. 92, ©2013 American Chemical Society.

The Bohr radius of CuInS_2_ is *ca.* 4.1 nm and thus CIS undergoes quantum confinement when nanocrystals are below 8.2 nm. This allows CuInS_2_ to absorb the entire visible region of the solar spectrum by control of the size of the nanocrystals used (Fig. 8).^92^

**Fig. 9:**
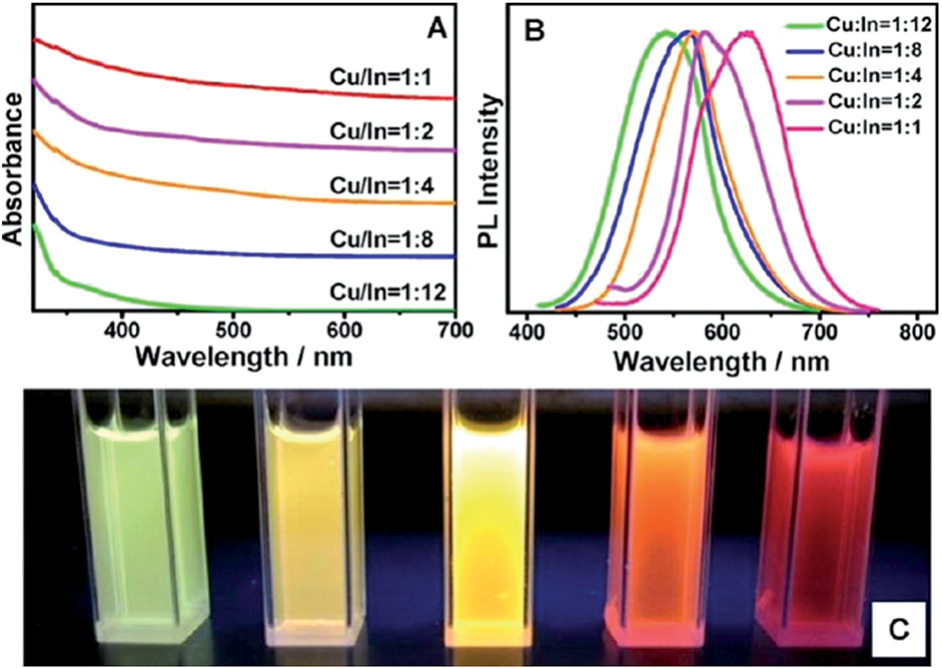
(a) Absorption and (b) fluorescence spectra of CIS nanoparticles with different Cu : In ratio and (c) a picture of the corresponding solutions under UV irradiation. Reprinted with permission from ref. 94, ©2013 American Chemical Society.

As previously described, the composition can be varied from the ideal 1 : 1 : 2 of CuInS_2_. Altering the Cu : In ratio causes the position of the valence band to change as it is composed of S-3p and Cu-3d orbitals. In copper poor nanocrystals the valence band falls and the bandgap widens (Fig. 9).^94^

In the same manner as CuInS2, CuInSe2 can also be an n- or p-type semiconductor depending on the abundances of In or Se.^95^ With a band gap of 1.02 eV, it has sub-optimal absorption characteristics with respect to the AM 1.5 solar emission spectrum in the bulk as-compared with CuInS_2_ but, on the other hand, has a very high absorption coefficient of 10^5^ cm^–1^ making it a good candidate for PV devices.^96^ As with CuInS_2_, the band gap of CuInSe_2_ can be controlled by altering the size and composition of the nanocrystals. CuInSe_2_ also undergoes strong quantum confinement compared to other copper based ternary metal chalcogenides, ranging from 1.0 eV for 6 nm particles to 3.2 eV at 1 nm particle diameter.^82^

Copper gallium selenide is also a I–III–VI_2_ ternary metal chalcogenide with the chalcopyrite structure. It possess a direct band gap of 1.66 eV and possesses a high optical absorption coefficient (10^5^ cm^–1^).^97^ The use of CuGaSe_2_ in photovoltaics has been hindered by the difficulty in producing a single phase material. In response to this there have been new synthetic routes to the formation of phase pure CuGaSe_2_ through colloidal routes.^98,99^

Unlike CuGaSe_2_, CuGaS_2_ has undergone far less investigation due to greater difficulty in the growth of a single phase. Bulk CuGaS_2_ possesses a direct band gap of 2.49 eV allowing for use in the visible (green) region of the electromagnetic spectrum.^100^ As with CIS there is a tendency to form intrinsic defects (caused by Cu vacancies and Ga substitution of Cu atoms) which greatly influence the observed properties of the material produced.^101^

The antimony analogue chalcostibite (CuSbS_2_) is a relatively under-studied compound, though has an appropriate direct band gap of 1.38–1.50 eV.^102–104^ It has the added benefit of composed of earth abundant and non-toxic elements, though phase pure CuSbS_2_ is hard to achieve owing to contamination of other copper antimony sulfide phases or binary impurities such as Cu_2_S and Sb_2_S_3_.^105^

For the I–III–VI_2_ chalcopyrite-type compounds described above, copper has been exchanged for silver as AgInS_2_,^106^ AgInSe_2_,^107^ AgGaS_2_,^108^ and AgGaSe_2_.^109^ The silver analogues have similar properties to the Cu compounds, but can be synthesised under milder conditions.^106^ The replacement of cheap copper with relatively expensive silver is unlikely to aid the industrial uptake of this class of material. However, as the material is used in thin film form the total silver required is miniscule and the ability to form phase pure films is a significant advantage.

A very different class of compound, the transition metal chalcogenide perovskite has been identified by density functional theory (DFT) as being a target of interest.^110^ Ammonium lead halide perovskites have become an extremely highly studied area that shows great promise,^111–113^ but the presence of toxic Pb is a concern for widespread uptake. Sun *et al.*, have proposed CaTiS_3_, BaZrS_3_, BaZrSe_3_ and CaHfSe_3_ as potential candidates.^110^ Limited experimental work has been undertaken on these compounds, and a second DFT study has indicated that they might present significant synthetic challenges.^114^

## Quaternary

Quaternary transition metal chalcogenide systems, *i.e.* those with a general formula M_*x*_M′_*y*_M′′_*z*_E_*n*_ (M = transition metal, M′/M′′ = transition or other metal and E = S, Se or Te), are amongst the most challenging to synthesise, but offer the greatest potential for highest efficiencies.

There are two major quaternary systems that have been substantially studied: copper indium gallium selenide [CIGS, Cu(In,Ga)Se_2_] and copper zinc tin sulfide/selenide [CZTS, Cu_2_ZnSn(S,Se)_4_]. The CIGS system is an established technology that has seen commercial application, whilst the CZTS one remains at the R&D stage.

Chalcopyrite-based solar cells were first developed using CuInSe_2_ as the absorber material, which has a band gap of 1.04 eV. However, it was discovered that the band gap could be tuned by adding gallium in place of indium to a maximum of 1.68 eV (for CuGaSe_2_). Optimisation of devices has led to the conclusion that a Ga/(Ga + In) ratio of 0.25–0.35 (*i.e.* CuIn_0.75_Ga_0.25_Se_2_) gives devices with optimal power conversion efficiencies. This corresponds to a band gap of 1.10–1.25 eV.^115^

CIGS is one of the few transition metal chalcogenide photovoltaics to have been commercialised, with a number of companies marketing devices with >15% efficiency.^115^ It has a number of attractive properties, which include benign grain boundaries and a tolerant phase diagram that allows for a variety in composition whilst maintaining phase.^116^

**Fig. 10:**
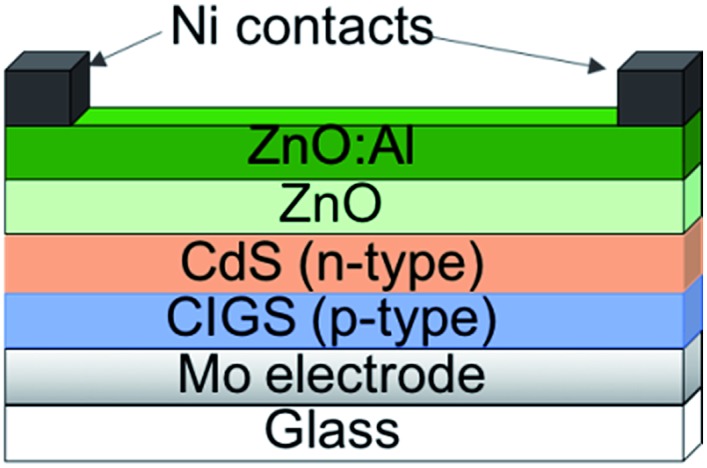
The typical architecture of a CIGS device.

In a typical CIGS device, Cu(In,Ga)Se_2_ is deposited on a Mo coated substrate, either by sputtering^116^ or through a solution process.^117^ At this stage Na or K are introduced as this improves the electronics of the device.^118,119^ On top of this a CdS buffer layer is grown (often by chemical bath deposition), followed by n-type ZnO/Al:ZnO transparent conducting windows. In some cases the CdS has been replaced with ZnS for a more environmentally friendly system.^120^ This leads to a typical device layout shown in Fig. 10.

The use of CIGS is undoubtedly a success in terms of efficiency, ease of manufacture and presence in the market, however, it has one major drawback which is shared by the ternary CIS (and its related family): availability of indium. The British Geological Survey ranks In as a ‘high supply risk’ in its 2015 Risk List.^12^ This has encouraged workers to turn to Cu_2_ZnSnS_4_ (CZTS) as an earth abundant, cheap and environmentally benign photovoltaic material.

**Fig. 11:**
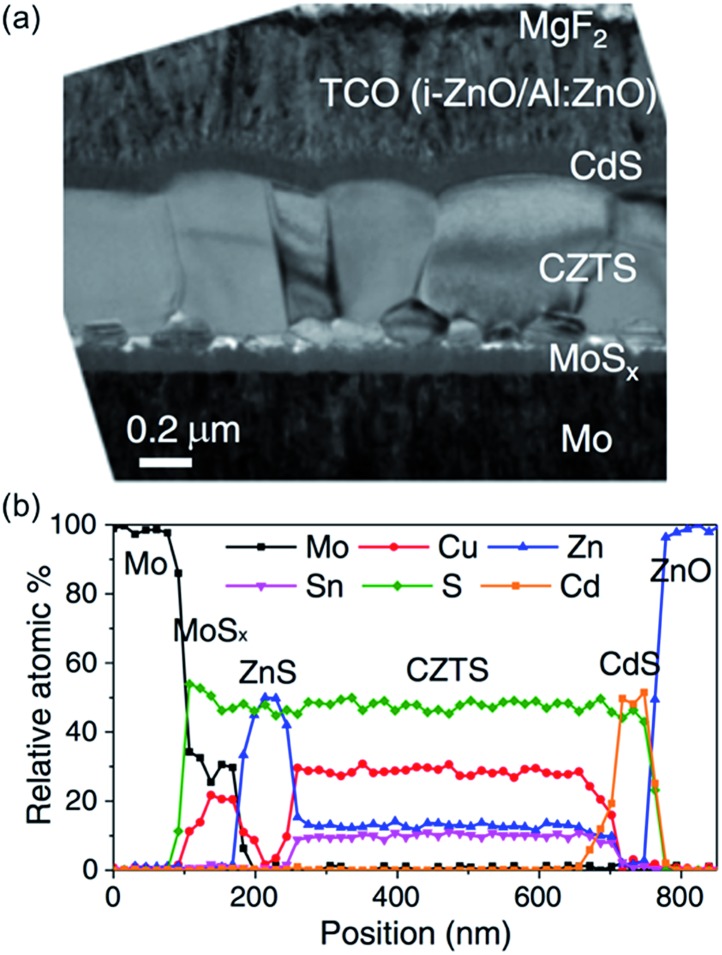
A cross-section of a typical CZTS device reveals the atomic composition of the different layers. (a) SEM image of a cross-section and (b) elemental composition determined by EDX as a function of position from the Mo contact. Reprinted with permission from ref. 129, ©2011 John Wiley & Sons, Ltd.

CZTS has a high absorption coefficient and a direct band gap of 1.45 eV which may be tuned by controlling the stoichiometry of the material.^121^ The current record efficiency is a very commendable 12.6%,^122^ indicating the great potential of this material. As with CIGS, it is often prepared through a sputtering or vapour deposition process, with a high temperature annealing step. There are two challenges that arise from this high temperature annealing step that must be overcome before CZTS becomes commercially viable. Firstly, the photoconversion efficiency of CZTS absorber layers is dependent upon the stoichiometry of the material, and during the annealing step volatile compounds such as SnS may be lost. This makes it difficult to control the composition of the target phase.^28,123–126^ Indeed, solar cells made from Cu poor films perform substantially better than those made from stoichiometric Cu_2_ZnSnS_4_.^123^ The second challenge is that the Mo electrode that CZTS is often deposited on can react with sulfur to form a MoS_2_ layer between electrode and absorber (Fig. 11), leading to a drastic loss of efficiency.^127–129^

There are further challenges in the fundamental materials science associated with CZTS as it can potentially exist in three stable phases (kesterite, stannite and a primitive mixed CuAu-like structure).^16,130,131^ The presence of these can influence the optical and electronic properties of the material. Additionally, the phase diagram for CZTS is not as tolerant as for CIGS and negative contamination with binary or ternary phases is likely.^121^

Despite these challenges, CZTS is one of the most viable transition metal chalcogenides for extensive industrial applications, owing to its cheap, abundant and non-toxic components. The highest efficiency devices of this class have come from solution processing methods, suggesting that this might be the best route to explore in the future.^122,132^

## Future outlook

The requirement for renewable energy sources remains a major research challenge at the present. Efficient and cheap photovoltaic materials are needed to tackle this challenge, and transition metal chalcogenides provide a viable route to this objective. Amongst ternary and quaternary systems some stand out candidates represent major targets for the future. CuInS_2_, Cu(In,Ga)S_2_ and Cu_2_ZnSnS_4_ are the three materials that are most likely to have a major impact. However, there are major concerns about the long term availability of indium and gallium, suggesting that alternatives must be sought. This leaves CZTS as the best hope. For it to fulfil its potential efforts must be directed to improve the manufacturing process, as this is a major source of the limits on its efficiency. Binary materials, such as Cu_2_S and FeS_2_, offer the greatest potential return when balancing theoretical energy conversion against cost. The challenge here is to produce large amounts of high quality, phase pure material.
